# ABUSE ACROSS THE LIFE COURSE: CONNECTIONS WITH ELDER ABUSE

**DOI:** 10.1093/geroni/igae098.0574

**Published:** 2024-12-31

**Authors:** Julie Miller, Lori Mars

**Affiliations:** Chair; Discussant

## Abstract

Likely conservative estimates suggest that 1 in every 10 Americans aged 60+ have experienced some form of elder abuse. As theoretical understandings of abuse in general have evolved, so too have conceptualizations of abuse at different stages of the life course, including in older adulthood. Questions remain, however, about ways in which abuse experienced earlier in the life course connect with subsequent experiences with elder abuse. This symposium brings together leading researchers and practitioners to explore abuse across the life course and connections with elder abuse. The first and second presentations in the symposium center experiences of elder abuse victims, with one presentation exploring patterns of abuse victimization across the lifespan and connections with prospective health outcomes in older adulthood, and another presentation focusing on experiences of financial exploitation among Holocaust survivors. The third and fourth presentations shift the perspective to emphasize experiences of people involved in the lives of elder abuse victims, including dementia caregivers who may be at greater risk for experiencing or perpetrating abuse once caregiving obligations begin and, in another case, concerned persons in the lives of elder abuse victims. Taken together, the presentations surface new insights and questions that locate elder abuse within a life course framework and connect abuse across the life course with elder abuse in particular. The symposium will include discussions of implications for research, practice, and policy.

